# SMV1 virus-induced CRISPR spacer acquisition from the conjugative plasmid pMGB1 in *Sulfolobus solfataricus* P2

**DOI:** 10.1042/BST20130196

**Published:** 2013-11-20

**Authors:** Susanne Erdmann, Shiraz A. Shah, Roger A. Garrett

**Affiliations:** *Archaea Centre, Department of Biology, University of Copenhagen, Ole Maaløes Vej 5, DK-2200 Copenhagen K, Denmark

**Keywords:** Archaea, clustered regularly interspaced short palindromic repeats spacer (CRISPR spacer), pMGB1, *Sulfolobus*, *Sulfolobus* monocaudavirus 1 (SMV1), transposable element, ATV, *Acidianus* two-tailed virus, Cas, CRISPR-associated, CRISPR, clustered regularly interspaced short palindromic repeats, crRNA, CRISPR RNA, IS, insertion sequence, PAM, protospacer-adjacent motif, p.i., post-infection, SIRV, *Sulfolobus islandicus* rod-shaped virus, SMV1, *Sulfolobus* monocaudavirus 1, SSV, *Sulfolobus* spindle-shaped virus, STIV, *Sulfolobus* turreted icosahedral virus, STSV1, *Sulfolobus tengchongensis* spindle-shaped virus 1

## Abstract

Organisms of the crenarchaeal order Sulfolobales carry complex CRISPR (clustered regularly interspaced short palindromic repeats) adaptive immune systems. These systems are modular and show extensive structural and functional diversity, especially in their interference complexes. The primary targets are an exceptional range of diverse viruses, many of which propagate stably within cells and follow lytic life cycles without producing cell lysis. These properties are consistent with the difficulty of activating CRISPR spacer uptake in the laboratory, but appear to conflict with the high complexity and diversity of the CRISPR immune systems that are found among the Sulfolobales. In the present article, we re-examine the first successful induction of archaeal spacer acquisition in our laboratory that occurred exclusively for the conjugative plasmid pMGB1 in *Sulfolobus solfataricus* P2 that was co-infected with the virus SMV1 (*Sulfolobus* monocaudavirus 1). Although we reaffirm that protospacer selection is essentially a random process with respect to the pMGB1 genome, we identified single spacer sequences specific for each of CRISPR loci C, D and E that, exceptionally, occurred in many sequenced clones. Moreover, the same sequence was reproducibly acquired for a given locus in independent experiments, consistent with it being the first protospacer to be selected. There was also a small protospacer bias (1.6:1) to the antisense strand of protein genes. In addition, new experiments demonstrated that spacer acquisition in the previously inactive CRISPR locus A could be induced on freeze–thawing of the infected cells, suggesting that environmental stress can facilitate activation. Coincidentally with spacer acquisition, a mobile OrfB element was deleted from pMGB1, suggesting that interplay can occur between spacer acquisition and transposition.

## Viruses and conjugative plasmids of the Sulfolobales

All known viruses infecting members of the Sulfolobales exhibit dsDNA genomes, either linear for the *Lipothrixviridae*, *Rudiviridae* and *Ampullaviridae* or circular for the *Bicaudaviridae*, *Fuselloviridae* and *Guttaviridae* and some unclassified viruses, with the genome sizes ranging from 14.7 kb [SSV2 (*Sulfolobus* spindle-shaped virus 2)] to 75 kb [STSV1 (*Sulfolobus tengchongensis* spindle-shaped virus 1)] [1–3] (Table 1). All of the viruses characterized, except for members of the family *Fuselloviridae*, exhibit lytic life cycles [4], but cell lysis has only been demonstrated for STIV (*Sulfolobus* turreted icosahedral virus) [5], SIRV2 (*Sulfolobus islandicus* rod-shaped virus 2) [6] and, less conclusively, for ATV (*Acidianus* two-tailed virus) when growth temperatures are reduced from 85°C to 75°C [7].

**Table 1:** Summary of the viruses and conjugative plasmids of the Sulfolobales Archaeal viral genomes are available at the European Nucleotide Archive (ENA) (http://www.ebi.ac.uk/genomes/archaealvirus.html).

Virus/plasmid type	Name	Genome	Size	Accession numbers
*Rudiviridae*	SIRV1, SIRV2, ARV1, SRV1, SMR1	Linear	27–35 kb	AJ414696, AJ344259, AJ875026, FM164764, JX944686
*Lipothrixviridae*	SIFV, AFV1, AFV2, AFV3, AFV6, AFV7, AFV8, AFV9	Linear	20–41 kb	AF440571, AJ567472, AJ854042, AM087120, AM087121, AM087122, AM087123, EU545650
*Fuselloviridae*	ASV1, SSV1, SSV2, SSV4, SSV5, SSV6, SSV7, SSV-K1, SSVRH, SMF1	Circular	15–21 kb	FJ870917, X07234, AY370762, EU030938, EU030939, FJ870915, FJ870916, AY423772, AY388628, KC618393
*Ampullaviridae*	ABV	Linear	23814 bp	EF432053
Turreted icosahedral	STIV, STIV2	Circular	16–18 kb	AY569307, GU080336
*Bicaudaviridae*	ATV	Circular	62730 bp	AJ888457
Monocaudaviruses	STSV1, STSV2, SMV1	Circular	65–76 kb	AJ783769, JQ287645, HG322870
*Guttaviridae*	SNDV	Circular	~20000 bp	Unsequenced [47]
Conjugative plasmids	pNOB8, pING1, pKEF9, pHVE14, pARN3, pARN4, pSOG1, pSOG2, pAH1, pMGB1	Circular	25–41 kb	AJ010405, AF233440, AJ748321, AJ748324, AJ748322, AJ748323, DQ335583, DQ335584, EU881703, HG008922

Members of the Sulfolobales also host a family of conjugative plasmids (Table 1). These plasmids encode a relatively simple conjugative apparatus comprising five to six core proteins that is archaea-specific and includes two large proteins carrying ATPase domains that may be distantly related to the bacterial conjugative proteins TraG and TrbE. The conjugative process is likely to involve the direct transfer of dsDNA [8–11]. Each plasmid also encodes an integrase, and remnants of conjugative plasmid genomes are commonly found in the chromosomes of Sulfolobales organisms where they may contribute to the capacity of some species to exchange chromosomal DNA [12,13].

## Diversity, complexity and mobility of *Sulfolobus* CRISPR systems

All characterized members of the Sulfolobales carry complex CRISPR (clustered regularly interspaced short palindromic repeats)-based immune systems and their properties are summarized for a few widely studied, and phylogenetically diverse, species in Table 2. Corresponding data for many *S. islandicus* species have been presented previously [14]. Extended CRISPR loci are generally present, often with more than 200 spacer-repeat units per cell [15,16], and many of the spacer sequences have been predicted by *in silico* analyses to derive either from specific viral families or the conjugative plasmid family [17–20]. Protospacers located on the invading genetic elements generally carry PAMs (protospacer-adjacent motifs) located at the protospacer end that becomes leader-proximal in a CRISPR array. Moreover, CRISPR loci tend to be associated with specific PAM sequences; for example, in *Sulfolobus solfataricus* P2, protospacers incorporated into loci C, D, E and F carry -CCN- PAM sequences, whereas loci A and B require -TCN- sequences (Table 2) where each PAM sequence is associated with a specific repeat sequence and leader region [18]. For Type I CRISPR systems, the PAM sequences have been implicated in both spacer acquisition and DNA strand-specific interference mechanisms. Given that the motif-recognition mechanisms are likely to differ, the acronyms SAM (spacer-acquisition motif) and TIM (target-interference motif) have been proposed recently to define the different sequence-recognition sites [21].

**Table 2:** Properties of CRISPR–Cas and CRISPR–Cmr systems of representative Sulfolobales members Numbers of chromosomal CRISPR loci and the total number of spacers are given for each organism together with the PAMs exhibited by their corresponding protospacers. An asterisk (*) indicates that the PAM sequence is based on comparison of only two sequences [21]. The total number of spacer-acquisition modules, *cas6* genes and interference modules are given and additional partial and potentially defective modules are enclosed by parentheses. Acquisition and interference modules are assigned to Type I or Type III CRISPR systems. Spacer-acquisition modules associated specifically with Type III interference modules are rare in archaea. A minus (−) indicates none present.

				Acquisition modules			Interference modules	
Organism	CRISPR loci	Spacers	PAM	I	III	*cas6* gene	I	III
*S. solfataricus* P2	6	415	CCN TCN	2	−	3	3	2 (2)
*S. islandicus* REY15A	2	206	CCN	1	−	1	1	2
*S. acidocaldarius* DSM639	5	223	GTN TCN	1 (1)	−	3	1	2
*S. tokodaii* 7	5	461	CCN TCN	2	−	3	2	3
*Acidianus hospitalis* W1	5	123	TCN CCN	(1)	1	2	1	1
*Metallosphaera sedula*	5	386	CCN ATTAN*	1 (1)	−	2	1	1

Both Type I and diverse Type III systems are generally present within cells and have the potential to generate different interference complexes targeting viral or plasmid DNA or their encoded RNAs. Subtype I-A and subtype III-B systems are widespread, whereas subtype I-D, subtype III-A and other unclassified Type III systems occur less frequently, and subtype I-B systems are found rarely [14,21,22]. Moreover, whereas subtype I-A spacer-acquisition and interference modules coexist in characterized Sulfolobales organisms, many Type III systems are limited to their interference modules, most of which are located distantly from CRISPR loci in host genomes and are inferred to utilize spacers acquired by Type I systems [14,21]. Evidence for such co-functionality of Type I and Type III systems was recently provided by the observation that a subtype III-B interference module of *S. islandicus* shared a crRNA (CRISPR RNA)-processing enzyme Cas6 (where Cas is CRISPR-associated) and single spacer-specific crRNAs with a coexisting subtype I-A system [22].

The presence of between one and four different variants of Type III interference systems encoded in closely related *Sulfolobus* strains suggests that they periodically exchange intercellularly [23]. This process may be facilitated by the CRISPR loci and *cas* gene cassettes of the Sulfolobales often being flanked by transposable elements and toxin–antitoxin gene pairs [15].

## Spacer acquisition in a subtype I-A_I_ system

Although considerable progress has been made in characterizing the processing mechanisms of archaeal CRISPR transcripts and the structures of the different interference complexes, we still have limited insight into mechanisms of spacer uptake into CRISPR arrays. Induction of spacer acquisition in laboratory strains of *Sulfolobus* species infected with viruses or carrying plasmids has proven very difficult. Numerous attempts were made in our laboratory, and in others, with single viral infections with no detectable spacer acquisition [24,24a]. This contrasts with the experience with bacteria where single phages or plasmids were shown to induce spacer uptake in subtype II-A CRISPR systems of *Streptococcus thermophilus* [25–27] and, more recently, in a subtype I-E system of *Escherichia coli* [28–30], albeit after first activating the CRISPR immune system by genetic manipulation.

This apparent contradiction may reflect, at least to some degree, differences between the virus–host relationships of archaea and bacteria. Most viruses infecting the Sulfolobales are lytic, but do not often cause cell lysis, although when cell lysis does occur, viruses are expelled via pyramid-shaped structures forming in the archaeal cell membrane [5,6]. In contrast, bacteriophages frequently cause cell lysis when extruding from their hosts. Members of the *Lipothrixviridae* and *Fuselloviridae* and the tailed fusiform STSV1 contain host lipids consistent with a budding mechanism for cellular extrusion which leaves the host cell intact [31–33], as has been observed for different eukaryal viruses [34]. Thus archaeal viruses tend to propagate in carrier-like states, and the cells are able to recover after extrusion of virus particles [31]. Therefore the host cell is not generally under an existential threat from the virus and the CRISPR system may not necessarily be activated, especially since spacer uptake is a costly process for the cell.

**Figure 1:**
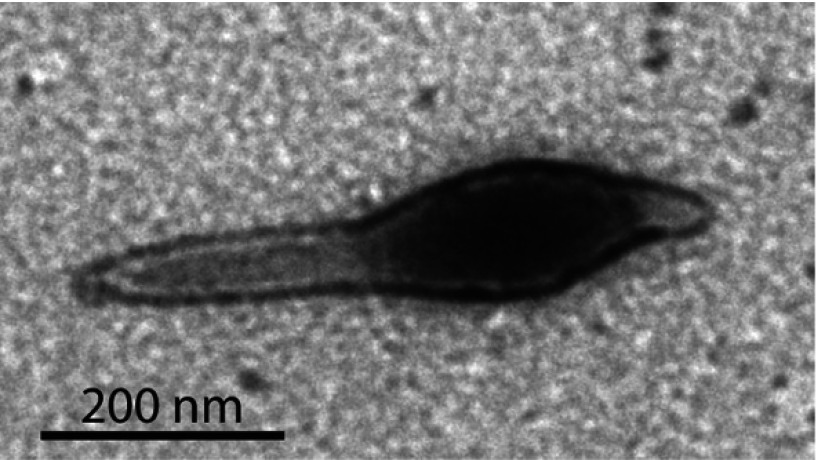
Electron micrograph of SMV1 Virus particles were adsorbed on to carbon-coated copper grids for 5 min, stained with 2% uranyl acetate, and examined using a JEM-1010 transmission electron microscope (Jeol).

The first successful archaeal spacer acquisition was observed on infecting *S. solfataricus* P2 with SMV1 (*Sulfolobus* monocaudavirus 1), a single-tailed fusiform virus isolated from Yellowstone National Park [24,24a] (Figure 1). However, unexpectedly, the new spacers did not derive from the virus; instead, they originated from a co-infecting conjugative plasmid pMGB1, a minor component in the viral preparation. Moreover, studies of isolated clones indicated that the *de novo* spacers produced resistance to the conjugative plasmid, but did not inhibit virus propagation. In these experiments, leader-proximal regions, and the whole loci for locus A (102 spacers), locus B (94 spacers), locus C (31 spacers), locus D (95 spacers), locus E (seven spacers) and locus F (88 spacers), were screened for spacer uptake by PCR amplification over a 70 day period p.i. (post-infection). Spacer uptake adjacent to the leaders was observed exclusively for loci C and D, and, exceptionally, it occurred throughout the smaller locus E via an altered insertion mechanism [24,24a].

A maximum of three new spacers were added in locus C, and four in locus D, even though spacer acquisition was an ongoing process in both loci over the 70 day period, as judged from the gradual increase in yield of the larger bands PCR-amplified from the leader-proximal regions. Only one new spacer was detected in clones of locus E and, in contrast with loci C and D, spacer uptake appeared to end at 16 days p.i. after which the proportion of larger PCR products amplified from locus E did not increase [24,24a].

Currently, we have no clear explanation for the exceptional spacer-acquisition mechanism of locus E. This locus is shared with *S. solfataricus* strains P1 and 98/2 and is also found in *S. islandicus* strain L.D.8.5 [35]. All four CRISPR loci carry a truncated leader region relative to the CRISPR leaders of other *Sulfolobus* strains. They share the 45 bp adjacent to the first repeat, but lack 13 bp from positions −46 to −59 that are present in the leaders of loci C and D of *S. solfataricus* P2 [23]. Possibly this leads to a reduction in the degree of specificity of binding the acquisition Cas protein complex. Another factor that could be important is that the first 34 nt of spacer 5 show a 26 nt sequence identity with the inverted leader region (positions −15 to −52) of CRISPR loci of *S. islandicus* M16.4 and related strains [16] that could, possibly, generate an alternative assembly site for the spacer-acquisition complex.

**Figure 2:**
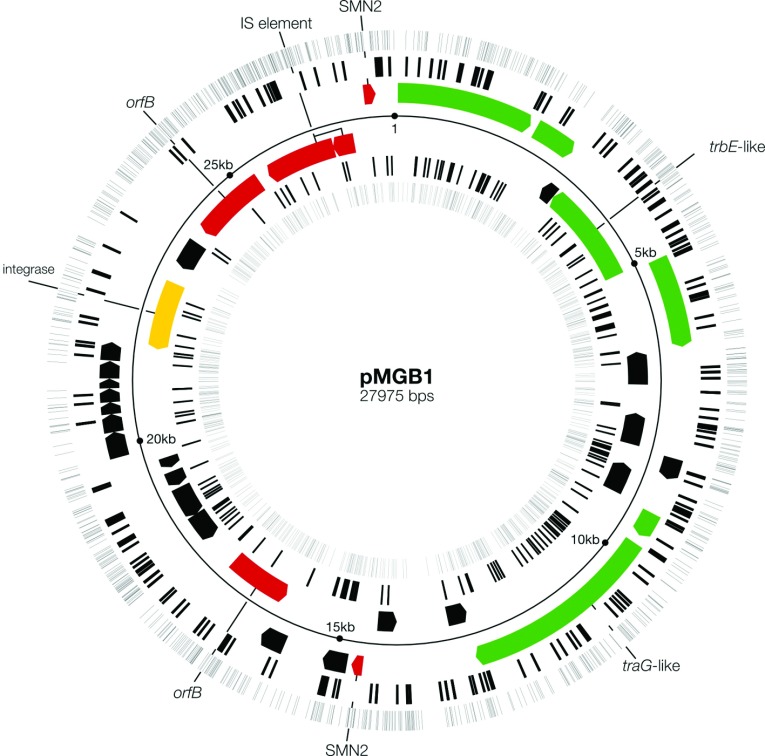
Distribution of -CCN- PAM sequences and experimentally determined protospacers on each DNA strand of the conjugative plasmid pMGB1 Spacers taken up in CRISPR loci C, D and E of *S. solfataricus* P2, in the presence of SMV1, are presented as black lines in concentric circles each denoting a DNA strand. The outermost and innermost circles show the distributions of all the potential CCN PAM sequences. Genes coloured green encode major components of the conjugative apparatus, yellow denotes the integrase and red indicates the mobile elements. The genome accession number at the European Nucleotide Archive is HG008922.

At the time of publication of the earlier article, complete genome sequences were not available for either SMV1 or pMGB1 [24,24a]. Thus the two genetic elements were incompletely annotated and several spacers could not be aligned on the genomes. A complete overview of the annotated pMGB1 genome and matching spacer locations was prepared after completion of the sequences and is shown in Figure 2. It demonstrates that the conjugation and integrase genes clustered in one half of the genome. A few mobile elements are also present, including an insertion sequence element IS200/IS650 and two non-identical OrfB elements. There are also two non-identical *Sulfolobus* mobile non-inverted repeat SMN2 elements, related to SMN1 [36] (Figure 2), that share 98% sequence identity and carry small putative ORFs. These elements are likely to be mobilized by the transposase encoded in the IS200 element. One of the SMN2 elements interrupts the *plrA* gene. PlrA is a DNA-binding protein, ubiquitous among *Sulfolobus* plasmids, that may have an important regulatory role such that its inactivation may alter the replication activity of pMGB1 [8–11].

## Activation of the subtype I-A_II_ system

The inactivity of subtype I-A_II_ CRISPR loci A and B in spacer acquisition on infection with SMV1 and pMGB1 contrasted with the hyperactivity observed for subtype I-A_I_ loci C, D and E. Loci A and B require the altered PAM sequence TCN, and both loci were shown to have been active in spacer uptake in closely related *S. solfataricus* strains in thermal hot-springs and, moreover, spacers matching both SMV1 and the distantly related bicaudavirus ATV, were identified in loci A and D [17,18,23,24,24a].

**Figure 3:**
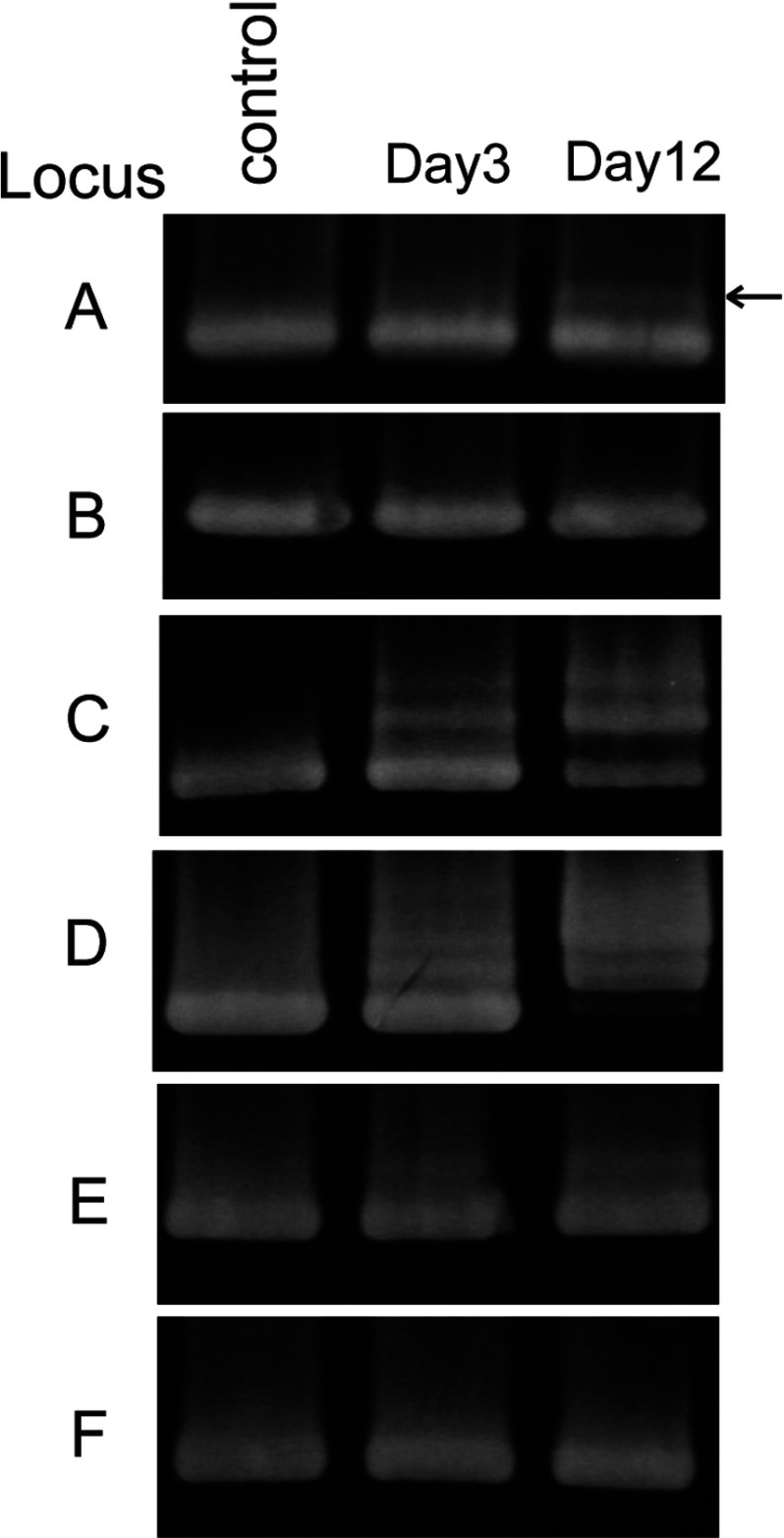
PCR amplification of the leader-proximal regions of CRISPR loci A to F The control sample shows the leader-proximal CRISPR region amplified from the uninfected wild-type *S. solfataricus* P2 strain. The larger amplified products visible for loci C, D and E from day 3, after reactivation of the culture from cells stored at −80°C, contain *de novo* spacers. A single upper band carrying *de novo* spacers, indicated by an arrow, was observed for locus A.

Later, we reactivated and examined frozen cells infected with SMV1 and pMGB1. Cells that had been actively undergoing spacer acquisition in loci C, D and E, 21 days p.i. were pelleted and stored at −80°C in medium containing 15% glycerol. After reactivating the culture, larger PCR products were still produced from the leader-proximal regions of loci C, D and E, yielding similar patterns to those reported previously [24,24a] (Figure 3). In addition, after approximately 6 days, a weak upper band appeared for locus A, but not for locus B (Figure 3). This PCR product from locus A was cloned and sequenced. Each clone was found to contain a single new spacer. In total, 11 *de novo* spacer sequences were obtained of which eight were unique and three were duplicated. Each matched pMGB1 perfectly and, with one exception, the PAM sequence was TCN (Table 3).

**Table 3:** *De novo* spacer sequences obtained from locus A after freeze–thawing pMGB1 genome positions are given for *de novo* spacers in locus A, where ‘c’ indicates a complementary strand match. The PCR-amplified upper band from the leader-proximal region of locus A (Figure 3) was produced using standard primers [24,24a], and sequences were obtained after cloning in *E. coli*.

pMGB1 sequence	PAM	Sequenced clones
824–860	TCT	1
3049–3083	TCC	3
5978–6017	TCA	1
13694–13732	TTT	1
c17875–17912	TCT	1
c17894–17928	TCT	1
18914–18949	TCT	2
23862–23900	TCT	1

Spacer insertion activity in locus A was low compared with loci C, D and E (Figure 3) and, in contrast with the earlier results from these loci when spacer uptake continued for at least 10 weeks [24,24a], it was detectable only from days 3 to 10 after reactivating the culture. This suggests that spacer uptake in locus A was activated as a stress response to the freeze–thawing process and was deactivated once the cells had recovered. This inference is supported by the fact that different types of stress can induce a lytic life cycle in different archaeal viruses, including the fusellovirus SSV1 and ATV [7,37]. The absence of new spacers in locus B may reflect an even lower spacer-acquisition activity of locus B with 94 spacers than locus A with 102 spacers.

## Distribution of selected protospacers on pMGB1

Early *in silico* protospacer prediction studies predated laboratory spacer-acquisition experiments and were limited to CRISPR spacers within available sequenced genomes of the Sulfolobales. The results of applying different statistical methods to these data were consistent with putative protospacers being distributed essentially randomly with respect to DNA strand, genes compared with intergenic regions, and conserved compared with less conserved genes [19,20,38]. However, the results did not necessarily reflect the *de novo* spacer-acquisition process because there may have been a selection for specific spacers post-acquisition. Initially, we analysed the *de novo* protospacer locations in partially assembled pMGB1 and inferred that they were also essentially randomly distributed with respect to DNA strand and to genes compared with intergenic regions [21,24,24a]. This inference is reinforced for the completely assembled pMGB1 sequence (Figure 2 and Table 4).

**Table 4:** Distribution of *de novo* spacer matches on the pMGB1 genome A total of 472 spacers were sequenced, of which 63 were duplicates. The forward strand corresponds to the outer strand in Figure 2. The antisense strand sequence is complementary to the mRNA transcript.

	Total pMGB1 protospacers	Proportion of protospacers (%)
Protospacer locations		
Forward (39.4% coding)	218	53
Reverse (43.7% coding)	191	47
Protein genes		
Antisense strand	228	56
Sense strand	144	35
Intergenic (16.9%)	37	9

However, when we analysed the protospacer distributions exclusively in protein genes of pMGB1, comparing antisense and sense strands, we detected a bias towards the antisense strand at a ratio of 1.6:1 (Table 4). This could possibly reflect a significant functional bias to mRNA targeting given that subtype III-B interference systems have been implicated in targeting RNA and transcribing DNA within hosts [22,39,40]. Furthermore, a re-examination of the earlier *in silico* analyses of predicted protospacers on genetic elements of the Sulfolobales also provided evidence of a weak bias to the antisense strand of protein-coding genes. However, the interpretation is complicated by the non-random strand distribution of the individual PAM sequences seen in pMGB1, and especially for the dominant CCT sequences which show a 1.4:1 bias towards the forward strand (Table 5), and requires further analysis of spacer-acquisition data from additional genetic elements.

**Table 5:** Analysis of PAM sequence distributions on pMGB1 Total numbers of each PAM sequence for the *de novo* spacers and the distribution of the theoretical PAM sequences on the two DNA strands of pMGB1.

	PAM				
	CCA	CCT	CCG	CCC	Total spacers/PAMs
*De novo* spacers	127	176	46	53	402
Forward strand PAMs	336	534	144	324	1338
Reverse strand PAMs	304	376	149	263	1092
Total PAMs	640	910	293	587	2430
Spacer/PAM ratio	0.2	0.19	0.16	0.09	

For subtype I-A systems of the Sulfolobales, there are many potential protospacers available for acquisition. A total of 1546 PAM sequences were identified, each of which can potentially produce multiple overlapping spacer sequences [24,24a] (Figure 2). Consequently, a few thousand unique spacers can be generated from pMGB1, consistent with the relatively few different duplicated sequences that were observed for the 409 unique *de novo* spacers. However, we did observe one significant bias. In each experiment, a single spacer was highly overrepresented for each CRISPR locus and the three different sequences are presented in Table 6. Moreover, for each locus, the same spacer sequence was dominant in independent experiments performed with a crude SMV1 preparation (experiment 1) and a purified SMV1 preparation (experiments 2 and 3), and, in addition, the individual spacers constituted a significant proportion of the total unique spacer sequences obtained for each locus (13–29%) (Table 6).

**Table 6:** Dominant single spacers acquired from pMGB1 by each of loci C, D and E Experiment 1 was performed with a crude SMV1 virus preparation, and experiments 2 and 3 were performed independently with a purified virus preparation [24,24a]. The total number of sequences containing the single spacer are given as a fraction of the total numbers of spacers cloned and sequenced in these specific experiments. The numbers derive from clones with both single and double spacer inserts for loci C and D, for which the sequence listed was invariably the first to be inserted into the CRISPR locus. pMGB1 genome sequence numbers are given where ‘c’ denotes a match to the reverse strand sequence.

			Single/total spacer sequences			
Locus	Multiple *de novo* spacer sequence (5′→3′)	pMGB1 genome	Experiment 1	Experiment 2	Experiment 3	Single spacer
C	CAGGAGGAACACTACTGGCAGCAATGCCAGAAATCAAAG	c15063–15101	4/54	18/209		8.3%
D	GAAATCAAAGGCCAAAAACCTACAGCGAAGGCGTAAAGGT	c15033–15072	36/115	23/103		27%
E	ATATTTCTCCATTACTCAAACGATATATAATGAAATCC	5762–5800	9/41	18/53	9/36	29%

More recently, another detailed spacer-acquisition analysis was performed on a subtype II-A system of *Streptococcus thermophilus* that was shown previously to be active in spacer uptake on infection with single phages [25,26]. A deep sequencing approach was applied to PCR-amplified *de novo* spacers deriving from protospacers adjoining up to 716 potential PAM sequences of the phage D2972. In contrast with the *Sulfolobus* subtype I-A results, they observed a very irregular distribution of protospacers on the genome [41]. They also found a few dominant sequences that could parallel the dominant single sequences seen for *Sulfolobus* (Table 6).

## Diversity of spacer-acquisition mechanisms used by different types of CRISPR systems

Most laboratory studies on the mechanisms of spacer acquisition have been performed on the genetically manipulated subtype I-E system of *E. coli*. This appears to be a relatively streamlined system involving primarily Cas1 and Cas2 proteins for the spacer uptake step that can function with a variety of degenerate PAM sequences that overlap by one nucleotide with the protospacer (or CRISPR repeat) [28–30,42]. Moreover, evidence has been provided for DNA strand-specific origin of multiple *de novo* spacers within a CRISPR locus of a single clone [29]. Additionally, it was proposed that the initial acquisition activity may be ‘primed’ by older spacers within a CRISPR array, producing crRNAs that can anneal partially to the invading virus [43]. Moreover, evidence was found for coupling of the interference and spacer-acquisition reactions [29].

*Sulfolobus* subtype I-A acquisition differs in several basic respects from the above. First, Cas4 has been strongly implicated in the acquisition stage and it has been shown to generate 5′-overhangs on DNA fragments compatible with a spacer uptake mechanism [23,40]. Possibly a co-functional host homologue, such as a RecB protein, fulfils this role in CRISPR systems lacking Cas4. Secondly, there is a stringent requirement for specific dinucleotide PAM sequences: CCN and TCN for the subtype I-A_I_ and subtype I-A_II_ subsystems respectively [21]. Thirdly, our data provide no evidence for strand directionality of spacers taken up within a given CRISPR locus of a single clone. Examination of 50 sequences from randomly selected single clones carrying two *de novo* pMGB1 spacers in either locus C or locus D showed that only 50% (25) were unidirectional. Finally, two apparently different spacer addition mechanisms have been characterized exclusively for *Sulfolobus.* Thus specific addition occurred at the leader-adjacent repeat as in *S. thermophilus* and *E. coli* and, in addition, at repeats located throughout CRISPR locus E of *S. solfataricus* P2. However, it remains to be demonstrated how commonly the latter mechanism operates. At present, there is no evidence for spacer-acquisition priming in *Sulfolobus*, although we cannot eliminate the possibility that the multiple host spacers matching SMV1 activate spacer acquisition which is then directed exclusively to pMGB1. There is also currently no direct evidence for coupling between the interference and spacer-acquisition reactions in Sulfolobales organisms.

We still know relatively little about the disparate Type III CRISPR systems. They generally occur in multiple diverse copies per cell (Table 2) and their primary cellular targets remain obscure. Subtype III-B systems have been shown to specifically degrade antisense CRISPR RNA *in vivo* in the crenarchaeon *Pyrococcus furiosus* and to target crRNA-complementary RNAs *in vitro* for *Sulfolobus* and, potentially, they can target and degrade any mRNA, non-coding RNA or antisense CRISPR RNA [39,40]. They are mainly encoded as discrete interference modules, separate from CRISPR loci, and the extent to which they influence spacer uptake remains unclear given that very few Type III interference gene cassettes are coupled directly with spacer-acquisition gene cassettes (Table 2). Moreover, it has been demonstrated experimentally that a subtype III-B module of *S. islandicus* can co-function with a CRISPR locus and a Cas6-processing enzyme associated with a subtype I-A system [22]. Currently, there is no evidence for subtype III-B systems acquiring spacers from, or targeting, RNA viruses, although, given their potentially widespread presence in terrestrial hot springs, this possibility cannot be eliminated [44].

## Links between the CRISPR-immune response and mobile elements

In the previous study, working with partially assembled genomes of the SMV1 virus and pMGB1, no *de novo* spacer matches to SMV1 were identified [24,24a]. However, on completion of the genome sequences, an IS element of the IS200/IS605 family, classified previously as ISC1467 [45], that was present in a pMGB1 contig, was also found in the completed SMV1 genome and showed 97% sequence identity (Table 7). When we reanalysed the 24 *de novo* pMGB1 spacers matching this element, we found that they all matched the viral element to some degree, with 12 showing perfect matches. The *S. solfataricus* P2 host also carries copies of closely related IS elements, but only one nearly identical sequence was found carrying a single mismatch (Table 7). The IS elements of both plasmid and virus appeared to be stable judging by the uniform sequence coverage, and, moreover, PCR amplification of their genomic locations did not reveal any loss of the IS element. Previously, eight spacer sequences in the CRISPR loci of *S. solfataricus* P2 were identified that matched perfectly to SMV1, one located in locus A (spacer 38) and seven in locus D (spacers 24, 26, 34, 35, 37, 39 and 40) and each exhibiting cognate PAM sequences [24,24a]. We therefore infer that the uptake of additional *de novo* spacers matching the IS element would be unlikely to inhibit virus propagation. pMGB1 also carries two non-identical copies of the SMN2 MITE (miniature inverted repeat transposable element)-like element (Figure 2) which show 89–91% identity with the two identical SMV1 SMN2 copies, but only one of the pMGB1 elements yielded a single perfectly matching spacer.

**Table 7:** Spacers matching IS200/IS605 in pMGB1 and SMV1 and the host *S. solfataricus* P2 *De novo* spacers matching the IS element for pMGB1, SMV1 and *S. solfataricus* P2 are listed. For each clone, the number refers to the experiment, C, D or E denotes the CRISPR locus which is followed by the sequence number. The number of mismatching nucleotides are given for pMGB1 and SMV1 and the host that carries multiple copies of closely related IS elements. A minus (−) indicates no match.

Spacers	Sequence (5′→3′)	pMGB1 match	SMV1 match	P2 match
Locus C				
1C38	TAGTTTAAAAGCCTTATCCCGTCCCTAGAAGGGGCGAG	0	4	5
1C49	CAACGTAGACAGTTGCTAAGTTTACTATCCCTAGGTCTAT	0	0	−
2C10	GTGGAAGTCCAGAATGACGTGAAAGCTGAAGGCAAACT	0	2	−
2C53	CACTCCATTCGTCCAGCGGTAGACCGCGGGCTGGGCCTT	0	2	−
2C54/3C1/Cm17	ATAGAGACCACGCTTAATACGCCCACGATGGTGGGCTT(C)	2	2	−
2C72	TTGAAAATATACCAGCTACCATCCTCAACGTAGACAG	0	0	−
Cm13	ATGATAAGCTTGCTCACACCCTTCTTCTTCAACTCCTCCAT	1	1	−
Locus D				
1D16/1E29	ACTCCTCCTCTGCTTATGCCTAGCCAAGGTTTTCTGGA	0	0	−
1D23	TTAATATTTGCTTGTACTTCTCGTATACATCCTTTTC	0	0	−
1D44	ACTGTTCCTCAATGTACTTCTTTATGGTCTCACTGGA	0	2	−
1D45	CTTTAGTGTATTCAGCTACCTCATTAGTTAGT	0	1	−
1D75	GAGCTAAGGAAGTACAAAAAGCTCTGGTCTAGAAGTTAT	0	2	−
2D13/1E9/2E23	ACATATCCCCGAGTCCCTAGGAGCTGGGAGCGGAGGG	0	0	−
2D27	AGTCCCTAGGAGCTGGGAGCGGAGGGCAACTCCCAG	0	1	−
2D38/C-R5	TTAACAGTATGGTAAGGAAGATAATGGAGGAGTTGAA	0	0	−
2D71	ATCCCTCACTGGGAGTTGCCCTCCGCTCCCAGCTCCTAG	0	1	−
Locus E				
1E18	CTCCATTCGTCCAGCGGTAGACCGCGGGCTGGGCCTT	0	0	−
2E7	TTTGCTATAATACTCGTACTGAGAGAGAACACTACCA	0	0	−
2E31	AGGTCTATGGAAGCCTTTAGTTTGCCTTCAGCTTTCA	0	0	−
3E2/A08-d65	ATGATAAGCTTGCTCACACCCTTCTTCTTCAACTCCTC	1	1	−
3E12	CATGATCTGACCTCCTCCCCGCCCTAAAGGGCGAGGGTT	0	5	7
3E13	CCCTAAAGGGCGAGGGTTCCCTTAGGGCGATTCATTGG	0	0	−
3E15	ACTAGCTATGAAGTGATGAAAATGAAGGCGGTAAACCG	0	0	1
3E16	AGATACTCGCCGTCATATCTAGCAAACTACTTCAAGGG	0	0	4

**Figure 4:**
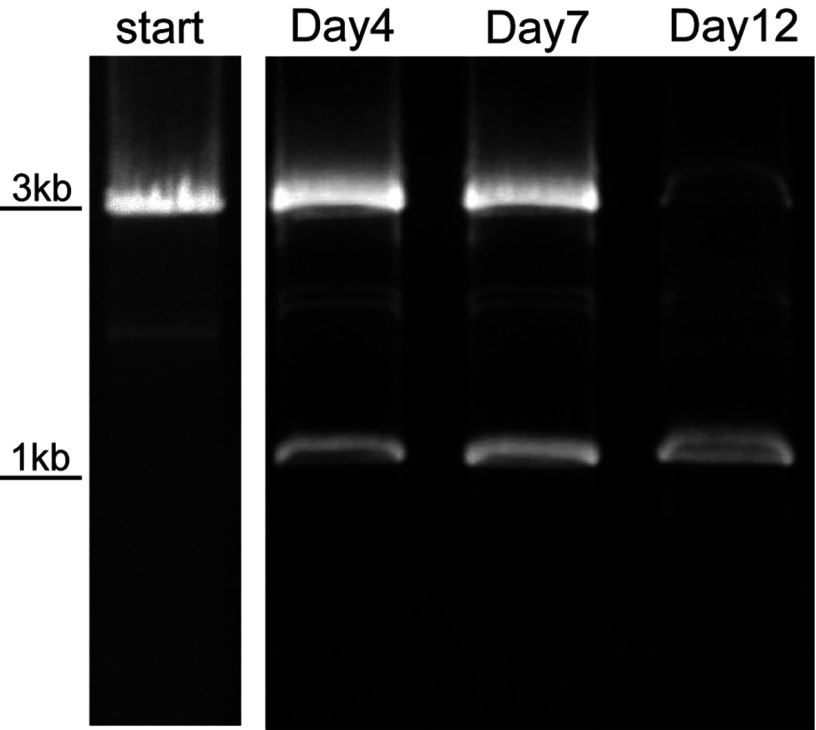
Transposition of an OrfB mobile element during spacer acquisition in *S. solfataricus* P2 PCR amplification of an OrfB element location in pMGB1 from a culture reactivated after storage at −80°C. Primers corresponding to pMGB1 genomic positions 22444–22459 and complement of 25758–25775 were used (see Figure 2). The OrfB element was completely lost from the reactivated culture over a 12-day period. *De novo* spacer acquisition was observed from day 3 (see Figure 3).

Direct evidence was found for transpositional activity occurring during spacer acquisition. pMGB1 carries two dissimilar mobile OrfB elements (Figure 2), of which one was absent from some sequenced clones of pMGB1, suggesting that it was actively transposing. PCR analyses of the OrfB location before and during spacer acquisition indicated that this OrfB element was gradually lost over a 12 day period, suggesting a link between spacer acquisition and transposition (Figure 4).

Interdependence of a CRISPR immune response and transpositional activity was also demonstrated earlier for *S. solfataricus* P2 during the interference stage. CRISPR systems were challenged by transformed plasmids carrying a protospacer sequence matching a specific CRISPR spacer and, in some surviving transformants which carried plasmids maintained under selection, the matching spacer had been specifically targeted by an IS element which destroyed the sequence match to the plasmid [46].

## Conclusions

Clearly, we are still in the early stages of understanding the mechanisms involved in spacer acquisition and maintenance in CRISPR loci and, as indicated above, there are significant differences in the molecular mechanisms of spacer acquisition operating for different types of CRISPR system and in phylogenetically diverse archaea and bacteria. All of our experiments with diverse single archaeal viral infections failed to initiate spacer acquisition in *S. solfataricus* P2, and we finally succeeded with a mixture of the tailed fusiform virus SMV1 and conjugative plasmid pMGB1 [24,24a]. Surprisingly, this virus propagated stably despite the presence of eight perfectly matching spacers in the host CRISPR loci, each with a cognate PAM sequence adjoining the putative protospacer on the viral DNA. However, currently we have no unambiguous explanation for this avoidance of interference. Moreover, the virus itself was resistant to spacer acquisition. We therefore concluded that other biological factors, including environmental stimuli, were required and this was supported by our successful activation of spacer uptake in locus A only after freezing and thawing the infected cells. A possible role for mobile elements is also supported by the coincidence of spacer acquisition from pMGB1 and the complete loss of an OrfB element.

The hyperactive uptake of immensely diverse spacers observed in the laboratory for a subtype I-A system of *Sulfolobus* [24,24a], and to a lesser degree for bacterial subtype I-E and II-A systems [28,29,41], is unlikely to occur in natural environments, including terrestrial hot springs, where a multitude of diverse organisms and genetic elements coexist at very low concentrations relative to the laboratory experiments. This inference is reinforced by comparative analyses of the CRISPR loci of closely related *S. solfataricus* strains isolated at different times over a 30-year period which show extensive sequence conservation of their CRISPR loci with very few *de novo* spacers added [17,18,23], consistent with CRISPR spacer contents changing very slowly in natural environments. We are currently investigating further the basis of the exceptional effects of the SMV1 virus on CRISPR spacer acquisition in different *Sulfolobus* species.
